# RAIN: RNA–protein Association and Interaction Networks

**DOI:** 10.1093/database/bax007

**Published:** 2017-02-10

doi: 10.1093/database/baw167

The corresponding author details for the above manuscript should read

*Corresponding author: Jan Gorodkin. Email: gorodkin@rth.dk

Correspondence may also be addressed to Email: lars.juhl.jensen@cpr.ku.dk

This has been corrected online, and the publisher apologizes for the error.

